# Interaction between smoking and functional polymorphism in the *TGFB1 *gene is associated with ischaemic heart disease and myocardial infarction in patients with rheumatoid arthritis: a cross-sectional study

**DOI:** 10.1186/ar3804

**Published:** 2012-04-18

**Authors:** Ying Chen, Peter T Dawes, Jon C Packham, Derek L Mattey

**Affiliations:** 1 Haywood Rheumatology Centre, Haywood Hospital, High Lane, Burslem, Stoke-on-Trent, Staffordshire, ST6 7AG, UK; 2 Institute of Science and Technology in Medicine, Keele University, Staffordshire, ST5 5BG, UK

## Abstract

**Introduction:**

Transforming growth factor-beta1 (TGF-beta1) is a pleiotropic cytokine that plays important roles in immunity and inflammation. Some studies have suggested that polymorphism in the *TGFB1 *gene is associated with heart disease in the general population. The purpose of the present study was to determine whether common single-nucleotide polymorphisms (SNP) in the *TGFB1 *gene are associated with ischaemic heart disease (IHD) and/or myocardial infarction (MI) in patients with rheumatoid arthritis (RA), and to investigate the influence of smoking on any association.

**Methods:**

PCR-based assays were used to determine the genotypes of *TGFB1 *SNPs including *TGFB1*-509 C/T (rs1800469, in the promoter region), +868 T/C (rs1800470, in exon 1) and +913 G/C (rs1800471, in exon 1) in 414 subjects with established RA. Genotyping for the +868 SNP was also carried out on a second study population of RA patients (*n *= 259) with early disease. Serum levels of TGF-beta1 were measured using a commercial ELISA kit. Smoking history and IHD/MI status were obtained on each patient. Associations with IHD/MI were assessed using contingency tables and logistic regression analyses.

**Results:**

The heterozygous genotype of *TGFB*+868 was associated with an increased risk of IHD (OR 2.14, 95% CI 1.30 - 3.55) and MI (OR 2.42, 95% CI 1.30-4.50), compared to the homozygous genotypes combined. Smoking was an independent risk for IHD and MI, and evidence of interaction between smoking and *TGFB*+868 was found. Multivariate analyses indicated that the strongest associations with IHD and MI were due to the combined effect of the *TGFB1*+868 TC genotype and smoking (OR 2.75, 95% CI 1.59-4.75; and OR 2.58 95% CI 1.33-4.99, respectively), independent of other cardiovascular risk factors. The association of the +868 TC genotype and evidence of +868 TC-smoking interaction with IHD were replicated in a second population of RA patients with early disease. Serum TGF-beta1 levels were not associated with *TGFB1 *genetic variations, smoking or IHD/MI status.

**Conclusions:**

Interaction between smoking and polymorphism in the *TGFB1 *gene may influence the risk of IHD and MI in patients with RA.

## Introduction

The excess risk of cardiovascular disease (CVD) associated with rheumatoid arthritis (RA) has long been recognized [1-4]. Among cardiovascular (CV) conditions, ischaemic heart disease (IHD), usually due to coronary artery disease (CAD), is the most common comorbidity in RA. A higher incidence of CAD in patients with RA in comparison with control subjects has been reported, and patients with RA are more likely to experience unrecognized myocardial infarction (MI) and sudden death [5]. Other studies have suggested that the increase of CV events in RA is due mainly to an excess of MI [6,7]. A number of risk factors for the development of CVD in RA have been established and these include classic risks such as smoking, hypertension, insulin resistance, body composition alterations, and RA characteristics such as autoantibodies, extra-articular disease, and increased inflammatory burden [1,2].

The genetic predisposition to CVD in RA has been the subject of an increasing number of studies in recent years. We and others have shown that certain *HLA-DRB1 *shared epitope alleles are associated with endothelial dysfunction and with the increased risk of CV events/mortality in RA [8-11]. Polymorphism in other genes in the HLA region, namely lymphotoxin A and tumor necrosis factor-alpha (*TNFA*) -308, has also been shown to be associated with CVD in RA [12,13]. Several polymorphisms in non-HLA genes have also been reported to be associated with CV conditions/events in RA [14-19].

Transforming growth factor-beta-1 (TGF-β1) is a multifunctional cytokine that plays an important role in a range of biological processes, including the modulation of immunity and inflammation, control of cellular proliferation, migration and differentiation, and regulation of tissue repair and extracellular matrix accumulation. In RA, it has been reported that TGF-β1 is produced in the synovial tissue and that enhanced expression of this cytokine is associated with remission of disease [20,21]. Furthermore, an animal model of arthritis revealed a relationship between TGF-β1 overexpression and disease reduction [22]. The role of TGF-β1 in the pathogenesis of atherosclerosis has long been the subject of debate. Inhibition of endogenous TGF-β signaling favors the development of atherosclerotic lesions [23], but a proatherogenic role of TGF-β1 is also suspected since it is able to promote fibrosis and to inhibit endothelial regeneration [24].

The *TGFB1 *gene is located on the long arm of chromosome 19 at position 13.2. Cambien and colleagues [25] described the common polymorphisms in Caucasians: -800 G/A (rs1800468) and -509 C/T (rs1800469) in the promoter region, a C insertion at position +72 in the non-translated region, +868 T/C (rs1800470) and +913 G/C (rs1800471) in exon 1, and +11929 C/T (rs1800472) in exon 5. Polymorphism in the *TGFB1 *gene has been associated with certain CV conditions (mainly MI) with different disease-associated single-nucleotide polymorphisms (SNPs) (-509, +868, and/or +913) in different studies [25-29]. However, other studies have reported no association [30-33]. An association between the *TGFB1*+868 SNP and hypertension has been demonstrated in RA [34], and we have reported an association of this SNP with mortality in RA [35]. To investigate the possible role of the *TGFB1 *gene in the development of IHD in RA, we have examined the association of selected SNPs (-509, +868, and +913) with the presence of IHD or previous MI in a cohort of patients recruited into a study of comorbidity in RA. The relationship between TGFB1 polymorphism and the circulating level of TGF-β1 was also investigated, as was the possibility of an interaction between smoking and the *TGFB1 *gene.

## Materials and methods

### Patients

This study was based on a cohort (*n *= 430) of patients who were consecutively recruited, were white Northern Europeans, had RA, and were residents of North Staffordshire, England. All patients had a diagnosis of RA and met the 1987 American College of Rheumatology criteria [36]. Written informed consent was provided by each patient in accordance with the Declaration of Helsinki. The research was approved by the North Staffordshire local research ethics committee. Sixteen (3.7%) samples were excluded from this report on the grounds that genotyping data or information about cigarette smoking was incomplete. Inclusion or exclusion of these samples made no significant difference to the associations found.

Most patients (93.5%) had been treated with one or more disease-modifying anti-rheumatic drugs (DMARDs). The majority were being treated with methotrexate (MTX), sulfasalazine (SSZ), or hydroxychloroquine. The commonest combination therapy of DMARD was MTX and SSZ. A small proportion of patients were being treated with steroids (9.7%) or cytotoxic drugs such as azathioprine or cyclophospamide (< 5%). Some patients (14.5%) were on a biologic agent (mainly etanercept and infliximab) at the time of recruitment.

Demographic data, including gender, age, weight, height, and occupation, were obtained for each patient. A core set of clinical and laboratory-based RA characteristics was recorded at recruitment. This included age at RA onset, disease duration, IgM rheumatoid factor (RF), anti-cyclic citrullinated peptide antibody, levels of C-reactive protein (CRP) and erythrocyte sedimentation rate, the Disease Activity Score using 28 joint counts (DAS28) [37], the Health Assessment Questionnaire [38], and the presence/absence of erosive and nodular disease. Information on current or past cigarette smoking was obtained from a questionnaire completed by each patient at recruitment, as described previously [17].

Evidence of cardiovascular disease (IHD, previous MI, heart failure, and so on) was obtained from a structured interview, review of the medical notes, and an inventory of current and cumulative medication. Furthermore, all patients underwent resting 12-lead electrocardiography (ECG). A diagnosis of IHD was based on the presence of angina pectoris, previous MI (physician-diagnosed) or evidence of CAD on the basis of angiography, functional testing, or previous revascularization procedures such as coronary artery bypass grafting. ECG was further used to identify possible unrecognized previous MI, as described previously [17]. Evidence of hypertension, hypercholesterolemia, and diabetes (type I and II) was obtained in a previous study on this cohort [17].

A second study population of patients with RA (*n *= 259, median age of 55.0 years) with early disease (median disease duration of 12 months) was used in a replication study of the association of *TGFB1 *polymorphism with IHD. Only patients with diagnosed definite RA (determined in follow-up in some cases) were included. In this cohort, the presence of IHD was determined by a structured interview and review of the medical notes. ECG measurements were not carried out in this group of patients.

### *TGFB1 *SNP typing

Leukocyte DNA was isolated from peripheral blood samples by using a Nucleon DNA extraction kit (GE Healthcare, Chalfont St Giles, Buckinghamshire, UK) in accordance with the instructions of the manufacturer. Polymerase chain reaction-restriction fragment length polymorphism (PCR-RFLP) analysis was applied to determine the genotypes of the *TGFB1*-509 SNP, and allelic-specific PCR systems were used to assess the genotypes of the *TGFB1*+868 and +913 polymorphisms. The genotyping methods for -509 and +868/+913 were described previously in [39] and [40], respectively. All primers were obtained from Sigma-Genosys (Haverhill, Suffolk, UK), and restriction enzyme was obtained from New England Biolabs (Hitchin, Hertfordshire, UK). All PCR amplification reactions were performed in a Flexigene thermal cycler (Techne (Cambridge) Limited, Cambridge, UK) by using a 96-well heating block.

### Quantification of circulating TGF-β1 level

Measurement of circulating TGF-β1 was performed on the serum samples by using a Duoset Human TGF-β1 enzyme-linked immunosorbent assay (ELISA) kit (R&D Systems, Minneapolis, MN, USA) and was read on a TiterTek Multiskan Plus MKII microplate reader (Flow Laboratories Ltd., Rickmansworth, Hertfordshire, UK). This assay measures the total TGF-β1 present (that is, the latent acid-activated molecule and any free, active TGF-β1). Sample and solution preparations and assay procedure were followed in accordance with the recommendations of the manufacturer.

### Statistical analysis

The relationship between *TGFB1 *genotypes and IHD/MI was initially analyzed by using contingency tables. Chi-squared *P *values are shown unadjusted and adjusted for multiple testing using the Bonferroni procedure. Meta-analysis was carried out by using MetaP [41] on the discovery and replication datasets (established and early RA, respectively). This combines the statistical association signals (*P *values) from independent study populations and takes into account the impacts of sample sizes and effect directions. A weighted Z-method (Stouffer's Z trend) was used to estimate the combined *P *value [42]. Multivariate logistic regression analysis was applied to investigate the independence of novel associations and to adjust for other possible confounders such as age, sex, hypertension, and diabetes. Evidence of interaction between smoking (ever-smoking) and *TGFB1 *polymorphism was assessed by examining for evidence of departure from additivity by using the methods of Rothman and Greenland [43]. By means of this approach, the attributable proportion due to interaction (AP) was calculated, together with 95% confidence interval (CI), as detailed by Andersson and colleagues [44]. The AP refers to the attributable proportion of disease that is due to interaction among individuals with both exposures. In the case of no biological interaction, AP equals 0, whereas an AP of 1.0 corresponds to complete additive interaction. This method has been suggested to be the most robust when using odds ratios (ORs) in place of relative risks [45].

By means of Haploview (version 4.2) [46], Hardy-Weinberg equilibrium (HWE) for the genotypic distributions of each polymorphism was tested with the chi-squared goodness-of-fit test. LD coefficient *D' *[47] and r^2 ^were used to estimate the strength of LD and allelic correlation between each pair of polymorphisms, respectively. The haplotype frequencies were estimated by using an expectation-maximization algorithm to determine the maximum-likelihood frequencies of multi-locus haplotypes [48]. Haplotypic association of *TGFB1 *genetic variation with serum TGF-β1 levels was investigated under regression-based models in HAPSTAT (version 3.0; Department of Biostatistics, University of North Carolina at Chapel Hill, NC, USA) [49].

Power calculations were performed by using an online power calculator [50]. As an example, for SNP *TGFB1*+868 (rs1800470), the study had 80% power to detect ORs of 1.7 or 2.6 for association of the risk allele with IHD at the 0.05 significance level, assuming recessive or dominant modes of inheritance, respectively. Multivariate logistic regression analyses were carried out by using the Number Cruncher Statistical System for Windows (NCSS 2000) (for stepwise selection analysis) or Stata (version 8.0) (for obtaining the covariance matrix for calculation of 95% CI for AP). The significance level was set at a *P *value of 0.05.

## Results

### Characteristics of patients with established RA

The characteristics of patients are displayed in Table 1. Evidence of IHD and previous MI was found in 20.8% and 12.6% of patients, respectively. Of the 52 patients with MI, 13 were identified on the basis of their ECG (Q-wave development in the absence of any conduction defect, suggesting full-thickness MI) in addition to the previously known cases. Patients with IHD or MI were older and were more likely to be male. No significant difference in disease duration between patients with or without IHD/MI was found.

**Table 1:** Selected demographic and clinical characteristics of rheumatoid arthritis patients stratified by the presence of ischaemic heart disease

Variable	All patients (*n *= 414)	Patients without IHD (*n *= 328)	Patients with IHD (*n *= 86)	*P *value^a^
Age, years	62.0 (54.8-69.0)	61.0 (54.0-68.0)	67.0 (58.0-72.3)	< 0.0001
Age of onset, years	50.0 (41.0-58.0)	49.0 (40.0-56.2)	54.9 (44.3-63.2)	0.0022
Duration, years	10.0 (3.6-18.0)	10.0 (3.0-18.0)	8.0 (4.0-18.0)	NS
Male/female	136/278	91/237	45/41	< 0.0001
Body mass index	27.3 (24.6-30.4)	27.3 (24.4-30.3)	27.4 (25.4-31.0)	NS
Rheumatoid factor	236/412 (57.3%)	178/326 (54.6%)	58/86 (67.4%)	0.032
Anti-CCP	305/402 (75.9%)	243/320 (75.9%)	62/82 (75.6%)	NS
ESR	20 (10-37)	18 (10-34)	26 (10-43.5)	NS
CRP (≥ 10 mg/L)	223/414 (53.9%)	162/328 (49.4%)	61/86 (70.9%)	0.0004
Nodules	54/414 (13.0%)	41/328 (12.5%)	13/86 (15.1%)	NS
Erosions	301/407 (74.0%)	243/322 (75.5%)	58/85 (68.2%)	NS
DAS28^b^	4.2 (1.4%)	4.1 (1.4%)	4.4 (1.4%)	NS
HAQ score	1.6 (1.0-2.0)	1.6 (0.9-2.0)	1.8 (1.3-2.3)	0.014
Ever-smoker	276/414 (66.7%)	205/328 (62.5%)	71/86 (82.6%)	0.0004
Current smoker	74/414 (17.9%)	58/328 (17.7%)	16/86 (18.6%)	NS
Previous MI	52/414 (12.6%)	-	52/86 (60.5%)	-
Hypertension	161/413 (39.0%)	110/327 (33.6%)	51/86 (59.3%)	< 0.0001
Hypercholesterolemia	68/414 (16.4%)	39/328 (11.9%)	29/86 (33.7%)	< 0.0001
Diabetes (I and II)	30/414 (7.3%)	13/328 (4.0%)	17/86 (19.8%)	< 0.0001
DMARD use	386/413 (93.5%)	306/327 (93.6%)	80/86 (93.0%)	NS
Methotrexate use	242/413 (58.6%)	204/327 (62.4%)	38/86 (44.2%)	0.0023
Steroid use	40/413 (9.7%)	28/327 (8.6%)	12/86 (14.0%)	NS
Biologic agent use	60/413 (14.5%)	53/327 (16.2%)	7/86 (8.1%)	NS
Serum TGF-β1 level, pg/mL	16,908 (12,744-21,428)	16,764 (12,422-21,772)	17,156 (13,435-20,533)	NS

Values other than *P *values are presented as number (percentage) or median (interquartile range). ^a^*P *values show significant differences between patients with and those without IHD (unadjusted); ^b^mean (standard deviation). Anti-CCP, anti-cyclic citrullinated peptide; CRP, C-reactive protein; DAS28, Disease Activity Score using 28 joint counts; DMARD, disease-modifying antirheumatic drug; ESR, erythrocyte sedimentation rate; HAQ, Health Assessment Questionnaire; IHD, ischemic heart disease; MI, myocardial infarction; NS, non-significant; TGF-β1, transforming growth factor-beta-1.

### Distributions of the *TGFB1 *SNP

Genotypes of the three SNPs were determined in 414 patients. The frequencies of *TGFB1 *genotypes were 49.3% (-509 CC), 44.2% (CT), and 6.5% (TT); 40.8% (+868 TT), 45.4% (TC), and 13.8% (CC); and 86.0% (+913 GG), 13.5% (GC), and 0.5% (CC). Genotypes of these polymorphisms were all distributed in accordance with a close fit to HWE. The allelic frequencies are shown in Additional Figure S1 of Additional file 1. These polymorphisms were strongly linked with each other, forming two major haplotypes - C-T-G (frequency 63.4%) and T-C-G (28.1%) - across the region.

### Serum TGF-β1 level

**Figure 1:**
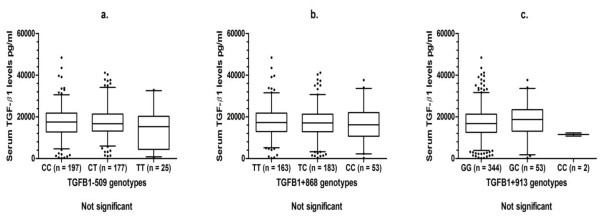
**Serum transforming growth factor-beta-1 (TGF-β1) levels stratified by TGFB1 single-nucleotide polymorphism (SNP) genotypes in patients with rheumatoid arthritis (RA)**. The boxplots show the median and interquartile range, and whiskers represent the 5th and 95th percentiles. Serum levels were determined in 399 subjects.

Serum TGF-β1 levels were determined in 399 (96.4%) patients and ranged from 183.2 to 48,360 pg/mL. The median (interquartile range) value was 16,908 pg/mL (12,744 to 21,428 pg/mL). Serum levels of TGF-β1 in each genotypic group of the studied *TGFB1 *polymorphisms are shown in Figure 1. These polymorphisms were not associated with serum TGF-β1 levels. Haplotype analysis indicated that no haplotype across these SNPs was associated with serum levels. The relationship of serum TGF-β1 levels with smoking and IHD/MI status was also investigated, but no significant associations were observed.

### Association of *TGFB1 *SNP with IHD and MI

The relationship between *TGFB1 *genotypes and the presence of IHD/MI, without adjustment for confounders, is shown in Table 2. There was no association between *TGFB1*-509 or *TGFB1*+913 genotypes and the presence of IHD/MI. In the case of *TGFB1*+868, the distribution of the data did not fit an additive, dominant, or recessive model for association with IHD, but a significantly increased risk was found for the heterozygous genotype compared with the two homozygous genotypes combined (Table 2). The heterozygous genotype also demonstrated an increased risk compared with each homozygous genotype individually (TC versus CC, OR = 2.52, 95% CI 1.10 to 5.79, *P *= 0.02, and TC versus TT, OR = 1.86, 95% CI 1.11 to 3.11, *P *= 0.02). An association of the +868 TC heterozygote with MI was also found, although an alternative dominant association of the T allele (TT + TC versus CC) could also be demonstrated. The association of the +868 TC heterozygote with IHD and MI remained significant after adjustment for multiple testing, although the association of the T allele with MI lost significance. Adjustment for age and sex in logistic regression analyses made little or no difference to the associations found (data not shown). No significant haplotypic association of *TGFB1 *SNP with IHD/MI was found.

**Table 2:** Frequency of ischaemic heart disease and myocardial infarction in rheumatoid arthritis patients stratified by *TGFB1 *SNP genotypes

	Ischemic heart disease		Myocardial infarction	
	Negative	Positive	Negative	Positive
*TGFB1*-509 (C/T)				
CC	165 (79.9%)	39 (19.1%)	181 (88.7%)	23 (11.3%)
CT	139 (76.0%)	44 (24.0%)	155 (84.7%)	28 (15.3%)
TT	24 (88.9%)	3 (11.1%)	26 (96.3%)	1 (3.7%)
CC + CT versus TT	OR = 1.92, 95% CI 0.61-6.04		OR = 2.70, 95% CI 0.51-14.38	
CT versus CC + TT	OR = 1.42, 95% CI 0.89-2.28		OR = 1.55, 95% CI 0.87-2.77	
*TGFB1*+868 (T/C)				
TT	141 (83.4%)	28 (16.6%)	151 (89.3%)	18 (10.7%)
TC	137 (72.9%)	51 (27.1%)	155 (82.4%)	33 (17.6%)
CC	50 (87.7%)	7 (12.3%)	56 (98.2%)	1 (1.8%)
TT + TC versus CC	OR = 1.92, 95% CI 0.85-4.30		OR = 6.32, 95% CI 1.22-32.89^a^	
TC versus TT + CC	OR = 2.02, 95% CI 1.25-3.27^b^		OR = 2.29, 95% CI 1.26-4.16^c^	
*TGFB1*+913 (G/C)				
GG	284 (79.8%)	72 (20.2%)	309 (86.8%)	47 (13.2%)
GC	42 (75.0%)	14 (25.0%)	51 (91.1%)	5 (8.9%)
CC	2 (100.0%)	0 (0.0%)	2 (100.0%)	0 (0.0%)
GG versus GC + CC	OR = 0.78, 95% CI 0.41-1.49		OR = 1.49, 95% CI 0.59-3.78	

'Negative' and 'positive' values are presented as number (percentage). ^a^*P *= 0.008, *P*_adj _= 0.072; ^b^*P *= 0.0037, *P*_adj _= 0.033; ^c^*P *= 0.005, *P*_adj _= 0.045. *P*_adj _are *P *values that have been adjusted for multiple testing (nine possible models) by using the Bonferroni procedure. CI, confidence interval; OR, odds ratio; SNP, single-nucleotide polymorphism; *TGFB1*, transforming growth factor-beta-1.

Since there is strong LD between *TGFB1*-509 and +868, the weaker signal from -509 may be due to the greater association of +868 with IHD and MI. This was supported by logistic regression analysis that contained both -509 (CT versus CC + TT) and +868 (TC versus TT + CC) together as independent variables and used forward stepwise selection to test for the primary risk factor. The associations involving *TGFB1*+868 maintained significance in relation to both IHD (OR = 2.14, 95% CI 1.30 to 3.55, *P *= 0.008) and MI (OR = 2.42, 95% CI 1.30 to 4.50, *P *= 0.007), whereas the association involving -509 disappeared in these models (adjusted for age and sex).

### Replication of association of the +868 TC genotype with IHD in patients with early RA

The association of the +868 SNP with IHD was examined in a second population of patients with early disease. Evidence of IHD was recorded in 27 out of 259 patients (10.4%). These patients were older (64.0 versus 54.0, *P *= 0.001) but had a disease duration (10 versus 12.0 months) similar to those without IHD and were more likely to be male (21.1% versus 12.3%, *P *= 0.06) and to have ever smoked (19.1% versus 10.6%, *P *= 0.1), although the differences were not significant. As in the cohort with established disease, the +868 TC heterozygote showed a significant association with IHD when compared with the homozygous genotypes combined (Table 3). Combining *P *values in a meta-analysis of the +868 TC association with IHD in the patients with established and early RA demonstrated a significant association (Stouffer's Z trend = 0.0003).

**Table 3:** Replication study: frequency of ischaemic heart disease in a second population of early rheumatoid arthritis patients stratified by *TGFB1*+868 SNP genotypes

	Ischemic heart disease	
*TGFB1*+868 (T/C)	Negative	Positive
TT	82 (93.2%)	6 (6.8%)
TC	118 (85.5%)	20 (14.5%)
CC	32 (97.0%)	1 (3.0%)
TT + TC versus CC	OR = 2.86, 95% CI 0.53-5.46	
TC versus TT + CC	OR = 2.64, 95% CI 1.10-6.34^a^	

'Negative' and 'positive' values are presented as number (percentage). ^a^*P *= 0.022. CI, confidence interval; OR, odds ratio; SNP, single-nucleotide polymorphism; *TGFB1*, transforming growth factor-beta-1.

### Association of *TGFB1*-smoking interaction with IHD and MI

Analysis was also carried out to investigate whether there was potential interaction of *TGFB1 *polymorphism with smoking relative to the occurrence of IHD/MI. Table 4 shows the occurrence of IHD and MI stratified by the combination of a *TGFB1 *heterozygous genotype (-509 CT and +868 TC) with ever having smoked. Evidence of interaction was tested on the basis of AP with 95% CI. For *TGFB1*-509, the values of AP (95% CI) demonstrated only non-significant results, although a borderline level was seen in relation to IHD. In contrast, for +868, AP showed a large proportion of effect due to interaction in relation to both IHD and MI, and the 95% CI indicated the significance of these results. Alternative models looking at the interaction between smoking and the -509 C allele or +868 T allele did not show evidence of significant interaction.

**Table 4:** Association of *TGFB1 *heterozygous genotypes with ischaemic heart disease and myocardial infarction in rheumatoid arthritis patients stratified by ever smoking

Ischemic heart disease				Myocardial infarction		
	Negative	Positive	OR (95% CI)	Negative	Positive	OR (95% CI)
Smoke/-509 CT						
-/-	65 (89.0%)	8 (11.0%)	1.0 (referent)	70 (95.9%)	3 (4.1%)	1.0 (referent)
-/+	58 (89.2%)	7 (10.8%)	0.99 (0.35-2.80)	61 (93.8%)	4 (6.2%)	1.47 (0.35-6.21)
+/-	124 (78.5%)	34 (21.5%)	2.14 (0.95-4.79)	137 (86.7%)	21 (13.3%)	3.15 (0.98-10.10)
+/+	81 (68.6%)	37 (31.4%)	3.55 (1.57-7.99) AP: 0.41 (-0.03-0.84)	94 (79.7%)	24 (20.3%)	5.22 (1.63-16.69) AP: 0.31 (-0.22-0.84)
Smoke/+868 TC						
-/-	68 (88.3%)	9 (11.7%)	1.0 (referent)	74 (96.1%)	3 (3.9%)	1.0 (referent)
-/+	55 (90.2%)	6 (9.8%)	0.84 (0.29-2.43)	57 (93.4%)	4 (6.6%)	1.67 (0.40-7.02)
+/-	123 (82.6%)	26 (17.4%)	1.55 (0.70-3.44)	133 (89.3%)	16 (10.7%)	2.63 (0.80-8.63)
+/+	82 (64.6%)	45 (35.4%)	3.98 (1.84-8.58) AP: 0.66 (0.34-0.97)	98 (77.2%)	29 (22.8%)	6.37 (2.02-20.10) AP: 0.49 (0.08-0.91)

'Negative' and 'positive' values are presented as number (percentage). AP, the attributable proportion due to interaction; CI, confidence interval; OR, odds ratio; *TGFB1*, transforming growth factor-beta-1.

Comparison within non-smokers suggested that the heterozygous genotypes of the *TGFB1 *gene alone had little effect on the risk for IHD and MI. However, patients who carried the heterozygous genotype and had ever smoked were at the highest risk for IHD and MI. The risk was significantly higher compared with that among patients who had ever smoked but did not carry these genotypes (for *TGFB1*+868 SNP in relation to IHD; smoke+/TC+ versus smoke+/TC-, OR = 2.57, 95% CI 1.48 to 4.47, *P *= 0.007; in relation to MI, OR = 2.42, 95% CI 1.26 to 4.67, *P *= 0.007).

### Replication of association of *TGFB1*+868-smoking interaction with IHD

An association of the +868 TC genotype with IHD in patients who had ever smoked but not in non-smokers was found in a separate population of patients with early RA (Table 5). Smoking status was available for 222 out of 259 patients in this RA cohort. As in established RA, the association with IHD in smokers was found only in patients who carried the *TGFB1*+868 TC genotype (smoke+/TC+ versus smoke+/TC-, OR = 3.55, 95% CI 1.18 to 10.67, *P *= 0.025). This was similar to the association seen when patients with the +868 TC/smoking combination were compared with all of the remaining patients (OR = 3.57, 95% CI 1.47 to 8.69, *P *= 0.003). Combining *P *values in a meta-analysis of the association of the +868 TC/smoking combination with IHD in the patients with established and early RA demonstrated a highly significant association (Stouffer's Z trend = 3.79 × 10^-7^).

**Table 5:** Replication study: association of the *TGFB1*+868 heterozygous genotype with ischaemic heart disease in early rheumatoid arthritis patients stratified by ever smoking

Ischemic heart disease			
Smoke/+868 TC	Negative	Positive	OR (95% CI)
-/-	25 (92.6%)	2 (7.4%)	1.0 (referent)
-/+	38 (95.0%)	2 (5.0%)	0.66 (0.11-4.10)
+/-	69 (94.5%)	4 (5.5%)	0.66 (0.13-3.31)
+/+	67 (81.7%)	15 (18.3%)	2.34 (0.57-9.61)
			AP: 0.84 (0.14-1.53)

'Negative' and 'positive' values are presented as number (percentage). AP, the attributable proportion due to interaction; CI, confidence interval; OR, odds ratio; *TGFB1*, transforming growth factor-beta-1.

### Multivariate associations with IHD and MI

We carried out multivariate logistic regression analysis by using models containing the *TGFB1*+868 TC-smoking interaction term as well as other known demographic and clinical risk factors. The multivariate association models obtained from forward selection analyses are shown in Table 6. Independent risk factors significantly associated with IHD included *TGFB1*+868 TC-smoking interaction, older age, male sex, CRP of at least 10 mg/L, hypercholesterolemia, and diabetes, whereas those associated with MI were *TGFB1*+868 TC-smoking interaction, male sex, CRP of at least 10 mg/L, RF+ (borderline association), hypertension, and hypercholesterolemia. Confirmation of the *TGFB1*+868 TC-smoking interaction with IHD independent of other risk factors was demonstrated in a separate population of patients with early RA (Table S1 of Additional file 2).

**Table 6:** Multivariate stepwise logistic regression analysis of variables associated with ischaemic heart disease and myocardial infarction

Ischemic heart disease (model 1^a^)				Myocardial infarction (model 2^b^)			
Variable	Regression coefficient	OR (95% CI)	*P *value	Variable	Regression coefficient	OR (95% CI)	*P *value
^c^Smoking+*TGFB1*+868TC	1.012	2.75 (1.59-4.75)	0.0003	^c^Smoking+*TGFB1*+868TC	0.948	2.58 (1.33-4.99)	0.0049
Age, per year	0.032	1.03 (1.01-1.06)	0.023	RF-positive	0.655	1.93 (0.93-3.99)	0.078
Male	0.744	2.10 (1.21-3.65)	0.0080	Male	0.972	2.64 (1.37-5.10)	0.0038
CRP ≥ 10 mg/L	1.030	2.80 (1.57-5.01)	0.0005	CRP ≥ 10 mg/L	0.810	2.25 (1.11-4.56)	0.025
Hypercholesterolemia	1.162	3.20 (1.69-6.04)	0.0003	Hypercholesterolemia	1.196	3.31 (1.58-6.91)	0.0014
^d^Diabetes	1.198	3.31 (1.36-8.06)	0.0083	Hypertension	1.009	2.74 (1.36-5.53)	0.0048

^a^Patients with ischemic heart disease (IHD) versus those without IHD; ^b^patients with myocardial infarction (MI) versus all non-MI patients; ^c^patients who have ever smoked and carry the TGFB1+868 TC genotype in comparison with all remaining patients; ^d^type I or type II diabetes. Forward stepwise selection was used to determine the variables most strongly associated with IHD and MI. Variables excluded by the stepwise procedure for IHD were disease duration, hypertension, erythrocyte sedimentation rate (ESR), rheumatoid factor (RF), anti-cyclic citrullinated peptide (anti-CCP), body mass index (BMI), methotrexate treatment, steroid treatment, erosive disease, and nodular disease. Variables excluded by the stepwise procedure for MI were age, disease duration, ESR, anti-CCP, BMI, diabetes, methotrexate treatment, steroid treatment, erosive disease, and nodular disease. CI, confidence interval; CRP, C-reactive protein; OR, odds ratio; RF, rheumatoid factor; *TGFB1*, transforming growth factor-beta-1.

Previously, using the same established RA cohort, we demonstrated that *VEGFA*-2578 A allele-smoking interaction was associated with an increased risk of IHD and MI [17]. The addition of the *VEGFA*-smoking interaction term into the above multivariate models demonstrated independent associations of both gene-smoking interaction combinations with IHD and MI (Table S2 of Additional file 3).

## Discussion

To our knowledge, this is the first study to investigate the involvement of the *TGFB1 *gene in the risk of CVD in patients with RA. The results have demonstrated an association of genetic variation in the *TGFB1 *gene with the occurrence of IHD, and MI in particular, and have further indicated a gene-smoking interaction relative to the association found. Similar results were found in RA populations with well-established and early disease.

According to univariate analyses, the *TGFB1*+868 SNP was associated with IHD and MI, and the -509 polymorphism showed a similar, though not significant, relationship. Multivariate logistic regression models containing both SNPs suggest that the trend involving -509 is not independent of +868 and may be due to LD. The +913 SNP was apparently neutral since it did not contribute measurable effects. These results are in line with those of Crobu and colleagues [27], who investigated the association of *TGFB1 *SNP with MI in young Italian patients. By means of univariate analysis only, the study showed that both -509 and +868 polymorphisms were associated with MI and that the stronger association occurred at position +868. Koch and colleagues [29] showed slightly different results in a study that included four *TGFB1 *SNPs (-509, +868, +913, and +11929) and found an association in males only. This study indicated that the primary MI-associated SNP was -509, although both -509 and +868 were associated with the disease in univariate analyses. A study from Japan indicated an association of *TGFB1*+868 with MI in men [26]. The above results suggest that the genetic locus tagged by *TGFB1*-509 or +868 is important in susceptibility to MI. Controversially, Cambien and colleagues [25] showed that *TGFB1*+913, rather than -509, or +868 provided the strongest signal in relation to the association with MI in Caucasian men from France or Northern Ireland.

It is not possible to say, on the basis of current data, which (if any) of the SNPs examined has a causal relationship. However, the *TGFB1*-509 SNP, in the promoter region of the gene, does not lie within a known regulatory sequence and so is unlikely to play a role in influencing the expression of TGF-β1. In contrast, the *TGFB1*+868 SNP encodes an amino acid change (Leu/Pro) in the signal peptide that is involved in export of the pre-proprotein across membranes of the endoplasmic reticulum [51] and so this SNP may be associated with the transportation or localization of TGF-β1 or both. A transfection study in HeLa cells indicated that the allele (C) encoding Pro 10 is associated with increased rates of TGF-β1 secretion and that the +868 SNP alone has a functional effect independent of any effect of the -509 polymorphism [52]. Previous studies have also reported that serum TGF-β1 levels are higher for Pro 10 homozygotes than Leu 10 homozygotes [26,53].

In the present study, we found no association of the *TGFB1*+868 or other SNPs with serum levels of TGF-β1. Therefore, we speculate that the genetic association seen with IHD/MI may be attributable to changes involving the processing or activation of TGF-β1 or both. TGF-β1 is secreted in a latent complex in which mature TGF-β1 dimers are associated with dimers of pro-peptide, termed the latency-associated peptide (LAP) [54]. Growth factor activation requires the release of TGF-β1 from its LAP. One possible explanation for the heterozygous association observed is that dimers of the pre-proprotein formed by a combination of wild-type and variant protein (+878 Leu/Pro 'heterodimer') have altered functional characteristics compared with either Leu/Leu or Pro/Pro homodimers. This involves the concept of 'molecular heterosis', which occurs when the heterozygote for a genetic polymorphism shows a significantly greater or lesser effect for a trait than the homozygotes. Heterosis is believed to be common in humans and can be gene-, phenotype-, gender-, and organ-specific [55].

Our data in patients with RA differed from those of studies in general Caucasian populations in which it has been suggested that the T allele at -509 or the C allele at +868 or both were the risk alleles in a dominant [27] or recessive [29] fashion. Data from Japan, however, showed that the T allele at +868 was associated with MI [26]. Interestingly, this is closer to our observation in patients with RA, although in this case a combination of the T and C alleles appears to provide the greatest risk. The regulation and activation of TGF-β1 may be different between the general population and individuals with certain disease conditions (particularly, autoimmune disease in which TGF-β1 plays an important role), and the potential for unique associations occurs in specific diseases. Furthermore, additional risk factors associated with IHD/MI in RA (for example, autoantibodies and increased inflammation) may contribute to divergent results between study populations.

It is particularly noteworthy that, in the absence of smoking, the *TGFB1*+868 heterozygous genotype did not confer an increased risk of IHD or MI. A gene-smoking interaction thus appears essential for the *TGFB1 *gene to play a role. The 'excess' risks for IHD and MI directly attributed to this interaction were approximately 65% and approximately 50%, respectively. The mechanism involved is unknown, although it is known that smoking affects cell-mediated and humoral immune responses and is associated with both release and inhibition of pro-inflammatory and anti-inflammatory mediators [56]. Recently, in chronic obstructive pulmonary disease, the oxidative effects of smoking (reactive oxygen species) were found to be associated with the activation of TGF-β [57]. Whether this effect also exists in CVD remains to be investigated.

Several publications have suggested an association of *TGFB1 *polymorphisms with CVD (mainly MI) [25-29], but, as far as we are aware, none has investigated the interaction between smoking and *TGFB1 *polymorphisms. It is interesting that, in some previous studies in the general population [25,26,29], the association of *TGFB1 *polymorphism with MI was observed among males only. It is possible that the previously reported lack of association among women is due to the lower frequency of female smokers in the general population and the naturally low incidence of MI for females. In the patients with RA in this study, interaction of *TGFB1*+868 with smoking has an effect in both genders (Table S3 of Additional file 4).

There are several limitations to this study. First, the number of patients with IHD or MI or both was relatively small, although we were able to replicate the findings in two separate RA cohorts with established and early RA. Further studies, preferably with a larger number of cases, will be needed to confirm these results. The second limitation is the possibility that some patients with silent, non-full-thickness, or atypical MI were missed. However, the MI incidence reported in our RA population is comparable to that of other studies [5,12]. Another limitation was the absence of a control population, and so it was not possible to show whether the association was RA-specific. Finally, the study was cross-sectional in design, and so no time course relationship was investigated, and it was possible to assess only those subjects who had survived previous events (for example, cardiac events and stroke).

## Conclusions

We have demonstrated an association of polymorphism in the *TGFB1 *gene with IHD and MI in RA. The strongest association appears to be with the heterozygous genotype of the +868 SNP and is dependent on an interaction with smoking. The association did not appear to be related to total serum levels of TGF-β1. If these findings are confirmed in further studies, this polymorphism may be useful for identifying those most at risk of IHD and MI among patients who have RA and who have smoked.

## Abbreviations

AP: attributable proportion due to interaction; CAD: coronary artery disease; CI: confidence interval; CRP: C-reactive protein; CV: cardiovascular; CVD: cardiovascular disease; DMARD: disease-modifying anti-rheumatic drug; ECG: electrocardiography; HLA: human leukocyte antigen; HWE: Hardy-Weinberg equilibrium; IHD: ischemic heart disease; LAP: latency-associated peptide; LD: linkage disequilibrium; MI: myocardial infarction; MTX: methotrexate; OR: odds ratio; PCR: polymerase chain reaction; RA: rheumatoid arthritis; RF: rheumatoid factor; SNP: single-nucleotide polymorphism; SSZ: sulphasalazine; *TGFB1*: transforming growth factor-beta-1; *VEGFA*: vascular endothelial growth factor-A.

## Competing interests

The authors declare that they have no competing interests.

## Authors' contributions

YC carried out the molecular genetic studies, TGF-β1 measurements, and statistical analysis and drafted the initial manuscript. PTD and JCP participated in the design of the study, recruitment of patients, and interpretation of ECG results. DLM conceived the study, participated in its design and coordination, and carried out statistical analysis and drafting of the final manuscript. All authors read and approved the final manuscript.

## Supplementary Material

Additional file 1 **Figure S1**. Allelic linkage between *TGFB1*-509, +868 and +913. Click here for file

Additional file 2 **Table S1**. Multivariate stepwise logistic regression analysis of variables associated with ischaemic heart disease in patients with early RA. Click here for file

Additional file 3 **Table S2**. Multivariate stepwise logistic regression analysis of variables associated with ischaemic heart disease and myocardial infarction in patients with established RA after inclusion of smoking+*VEGFA*-2578A interaction term. Click here for file

Additional file 4 **Table S3**. Association of *TGFB1*+868 heterozygous genotype and ever having smoked with ischaemic heart disease and myocardial infarction in RA patients stratified by sex. Click here for file
