# Supplementary material for: Interaction between smoking and functional polymorphism in the TGFB1 gene is associated with ischaemic heart disease and myocardial infarction in patients with rheumatoid arthritis: a cross-sectional study
Source: Arthritis Res Ther. 2012 Apr 18;14(2):R81. doi: 10.1186/ar3804 (PMC3446455; doi:10.1186/ar3804)
Supplement: Additional file 1 — Figure S1. Allelic linkage between TGFB1-509, +868 and +913. [file ar3804-S1.DOC]

**Figure S1.** Allelic linkage between TGFB1-509, +868 and +913


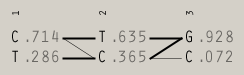


1, 2, 3 represent TGFB-509, +868 and +913, respectively. Numbers next to alleles show the frequencies of the alleles. Thick/thin lines represent the different strength of linkage, with thin lines showing the connection > 1.0% but ≤ 10.0%, while thick lines showing the connection > 10.0%.

LD coefficient (*D’*) and allelic correlation (r^2^) for pairs (-509, +868), (-509, +913) and (+868, +913) were *D*_(1, 2)_’ = 0.98, r_(1, 2)_^2^ = 0.66; *D*_(1, 3)_’ = 0.98, r_(1, 3)_^2^ = 0.03; *D*_(2, 3)_’ = 1.0, r_(2, 3)_^2^ = 0.13, respectively. A high *D’* and low r^2^ can occur if the alleles that tend to occur on the same haplotype have very different allele frequencies. Two major haplotypes, C-T-G (63.4%) and T-C-G (28.1%), were formed.
