# Supplementary material for: Interaction between smoking and functional polymorphism in the TGFB1 gene is associated with ischaemic heart disease and myocardial infarction in patients with rheumatoid arthritis: a cross-sectional study
Source: Arthritis Res Ther. 2012 Apr 18;14(2):R81. doi: 10.1186/ar3804 (PMC3446455; doi:10.1186/ar3804)
Supplement: Additional file 2 — Table S1. Multivariate stepwise logistic regression analysis of variables associated with ischaemic heart disease in patients with early RA. [file ar3804-S2.PDF]

Table S1. Multivariate stepwise logistic regression analysis of variables associated with ischaemic heart disease in patients with early RA

Variable	Regression coefficient	OR (95% CI)	p value
Hypertension	1.860	6.42 (2.28 – 18.09)	0.0004
[a]Smoking+TGFB1+868 TC	1.242	3.46 (1.32 – 9.04)	0.010
Age, per year	0.055	1.02 (1.01 -1.04)	0.011

[a]Patients who have ever smoked and carry the TGFB1+868 TC genotype, compared with all remaining patients. Forward stepwise selection was used to determine the variables most strongly associated with IHD. Variables excluded by the stepwise procedure for IHD were sex, disease duration, ESR, CRP, RF, anti-CCP, erosive disease and nodular disease.
