# Supplementary material for: Interaction between smoking and functional polymorphism in the TGFB1 gene is associated with ischaemic heart disease and myocardial infarction in patients with rheumatoid arthritis: a cross-sectional study
Source: Arthritis Res Ther. 2012 Apr 18;14(2):R81. doi: 10.1186/ar3804 (PMC3446455; doi:10.1186/ar3804)
Supplement: Additional file 3 — Table S2. Multivariate stepwise logistic regression analysis of variables associated with ischaemic heart disease and myocardial infarction in patients with established RA after inclusion of smoking+VEGFA-2578A interaction term. [file ar3804-S3.PDF]

Table S2. Multivariate stepwise logistic regression analysis of variables associated with ischaemic heart disease and myocardial infarction in patients with established RA after inclusion of smoking+VEGFA-2578A interaction term

Ischaemic Heart Disease (model 1[b])				Myocardial Infarction (model 2[c])			
Variable	Regression coefficient	OR (95% CI)	p value	Variable	Regression coefficient	OR (95% CI)	p value
[a]Smoking+VEGFA-2578 A	0.870	2.39 (1.31 – 4.36)	0.005	[a]Smoking+VEGFA-2578 A	1.344	3.83 (1.69 – 8.69)	0.001
Hypercholesterolaemia	1.182	3.26 (1.72 – 6.20)	0.0003	Hypercholesterolaemia	1.376	3.95 (1.90 – 8.24)	0.0002
CRP ≥ 10 mg/l	1.036	2.82 (1.57 – 5.06)	0.0005	Hypertension	0.927	2.52 (1.25 – 5.12)	0.010
[d]Diabetes	1.214	3.37 (1.36 – 8.34)	0.009	CRP ≥ 10 mg/l	0.920	2.51 (1.23 – 5.10)	0.011
Male	0.636	1.89 (1.08 – 3.31)	0.026	Male	0.769	2.16 (1.10 – 4.23)	0.025
[e]Smoking+TGFB1+868 TC	0.797	2.22 (1.26 – 3.92)	0.006	[e]Smoking+TGFB1+868 TC	0.734	2.08 (1.06 – 4.11)	0.034
Age, per year	0.028	1.03 (1.01 – 1.06)	0.049				

[a]Patients who have ever smoked and carry the VEGFA-2578 A allele, compared with all remaining patients; [b]Patients with IHD vs. without IHD; [c]Patients with MI vs. all non-MI patients; [d]Type I or type II diabetes; [e]Patients who have ever smoked and carry the TGFB1+868 TC genotype, compared with all remaining patients. Forward stepwise selection was used to determine the variables most strongly associated with IHD and MI. Variables excluded by the stepwise procedure for IHD were disease duration, hypertension, ESR, RF, anti-CCP, body mass index (BMI), methotrexate treatment, steroid treatment, erosive disease and nodular disease. Variables excluded by the stepwise procdure for MI were age, disease duration, ESR, RF, anti-CCP, BMI, diabetes, methotrexate treatment, steroid treatment, erosive disease and nodular disease.
