# Supplementary material for: Interaction between smoking and functional polymorphism in the TGFB1 gene is associated with ischaemic heart disease and myocardial infarction in patients with rheumatoid arthritis: a cross-sectional study
Source: Arthritis Res Ther. 2012 Apr 18;14(2):R81. doi: 10.1186/ar3804 (PMC3446455; doi:10.1186/ar3804)
Supplement: Additional file 4 — Table S3. Association of TGFB1+868 heterozygous genotype and ever having smoked with ischaemic heart disease and myocardial infarction in RA patients stratified by sex. [file ar3804-S4.PDF]

Table S3. Association of TGFB1+868 heterozygous genotype and ever having smoked with ischaemic heart disease and myocardial infarction in RA patients stratified by sex

	Ischaemic Heart Disease			Myocardial Infarction		
	Negative	Positive (%)	OR (95% CI)	Negative	Positive (%)	OR (95% CI)
Female (n = 278)						
Smoke/+868 TC						
-/-	54	8 (12.9)	1.0 (referent)	59	3 (4.8)	1.0 (referent)
-/+	48	5 (9.4)	0.73 (0.23 – 2.27)	50	3 (5.7)	1.18 (0.26 – 5.43)
+/-	82	11 (11.8)	0.89 (0.35 – 2..31)	87	6 (6.5)	1.26 (0.33 – 4.83)
+/+	53	17 (24.3)	2.10 (0.85 – 5.17)	60	10 (14.3)	2.95 (0.78 – 10.42)
			AP: 0.72 (0.17 – 1.27)			AP: 0.53 (-0.19 – 1.25)
Male (n = 136)						
Smoke/+868 TC						
-/-	14	1 (6.7)	1.0 (referent)	15	0 (0.0)	1.0 (referent)
-/+	7	1 (12.5)	1.93 (0.17 – 21.93)	7	1 (12.5)	6.20 (0.10 – 16.57)
+/-	41	15 (26.8)	3.61 (0.61 – 21.40)	46	6 (6.5)	7.0 (0.54 – 34.36)
+/+	29	28 (49.1)	9.34 (1.61 – 54.18)	38	19 (33.3)	15.70 (1.31 – 79.84)
			AP: 0.55 (0.07 – 1.02)			AP: 0.28 (-0.50 – 1.06)

Values are number (%); AP, the attributable proportion due to interaction.
